# A Nationwide Analysis: Memantine in the Treatment of Dementia in Hungary

**DOI:** 10.1192/j.eurpsy.2026.10668

**Published:** 2026-07-31

**Authors:** P. Halmos, D. Bereczki, B. Dobi, P. Vinnai, A. Ajtay, T. Kovács

**Affiliations:** 1 Doctoral College János Szentágothai Neurosciences Division; 2 Department of Neurology; 3 HUN-REN SE Neuroepidemiological Research Group, Semmelweis University, Budapest, Hungary

## Abstract

**Introduction:**

Dementia is a leading cause of dependency and disability, placing a substantial burden on patients, caregivers, healthcare, and social care systems. Considering its profound societal and economic impact, dementia represents a public health priority that calls for the development of national strategies to mitigate its future burden.

**Objectives:**

The aim of this retrospective cohort study was to provide a comprehensive overview of the current state of the healthcare system, placing special emphasis on anti-dementia drug (ADD) – especially memantine - utilisation patterns and their association with survival outcomes.

**Methods:**

Using the NEUROHUN database containing inpatient and outpatient care data as well as pharmacy reports for prescriptions submitted for reimbursement, we analyzed health data covering the entire population of Hungary between 2010 and 2020. Descriptive statistics were calculated to assess disease incidence and prevalence, followed by Kaplan–Meier survival analyses and Cox proportional hazard regression to evaluate survival outcomes. Sex, age at diagnosis, dementia subtypes, diagnosis switch, calculated adherence and persistence, as well as psychotropic drug use were also included as independent variables. All analyses were performed in R (version 4.5.1).

**Results:**

We identified 398,584 patients with a diagnosis of dementia between 2010 and 2020. The mean age at diagnosis was 75,22 years (SD = 12,110), and 63.7% of patients were female.

Of the total cohort, only 13.3 % (n = 53,070 ) of the patients were treated with at least one of the anti-dementia medications examined (memantine, donepezil or rivastigmine). Among the medication-fillers, 12757 (24.04 %) patients were treated with memantine only.

Kaplan–Meier analysis demonstrated that the untreated group had the shortest median survival ( 3.105 years, 95% CI: 3.083 - 3.129), whereas among patients on medication, those treated with memantine monotherapy had the shortest median survival (3.704 years, 95% CI: 3.606 - 3.784).

In the Multivariable Cox proportional hazards model, patients receiving a combination of memantine and donepezil had the lowest mortality risk (HR: 0.581,95% CI: [0.566-0.597]) compared to the untreated group.

**Conclusions:**

These findings suggest a high proportion of delayed diagnoses, as memantine is typically prescribed only in advanced dementia stages. Timely diagnosis—particularly in the era of emerging disease-modifying therapies—along with prevention programs and evidence-based healthcare planning is therefore essential.

**Disclosure of Interest:**

None Declared

